# Effects of Ultrasonic Introduced by L-Shaped Ceramic Sonotrodes on Microstructure and Macro-Segregation of 15t AA2219 Aluminum Alloy Ingot

**DOI:** 10.3390/ma12193162

**Published:** 2019-09-27

**Authors:** Tao Zeng, YaJun Zhou

**Affiliations:** 1 College of Mechanical and Electrical Engineering, Central South University, Changsha 410083, China; taozg1203@163.com; 2 National Key Laboratory of High Performance Complex Manufacturing, Central South University, Changsha 410083, China

**Keywords:** ingot solidification, L-shaped ceramic sonotrode, grain refinement

## Abstract

The effects of ultrasonic introduced by L-shaped sonotrodes made of high-temperature-resistant ceramic on the microstructure and macro-segregation of solidifying 15t AA2219 aluminum alloy ingots have been examined in the present study. The macroscopic morphology of the corrosion of the sonotrode has been observed. Grain refinement has been observed, the shape and size of the precipitated phase of the ingot were counted, and the degree of segregation along the transverse direction at 500 mm from the head of the ingot has been evaluated. The results reveal that the L-shaped ceramic ultrasonic introduction device can effectively avoid the erosion of high-temperature melt on the sonotrode and the heat radiation of the high-temperature heat flow to the transducer. Furthermore, the scanning electron microscope (SEM) and chemical composition detection results also indicate that the inter-dendritic micro-segregation of the equiaxed grains can be reduced, and the macro-segregation of the chemical composition of the ingot can be suppressed, and more homogeneous microstructures can be obtained when ultrasonic has been applied during solidification.

## 1. Introduction

AA2219 aluminum alloy is a heat treatable reinforced aluminum alloy with Al, Cu, and Mn as its main alloying elements. Because of its good high- and low-temperature mechanical properties, formability, machinability, and welding properties, it has been widely used in the aerospace industry, especially as the main material for the new generation of launch vehicle propellant tanks [1,2,3,4,5]. The original billet of the tank is a large-sized aluminum alloy ingot, which is difficult to form. It adopts the traditional casting method and is prone to defects such as loose shrinkage, coarse grain, serious segregation, and cracking of the structure, which extremely affect its performance and lead to its unstable production in batches [6,7].

Li Xiaoqian et al. [8,9] conducted a large number of casting experiments on 2XXX and 7XXX aluminum alloys using a straight-rod titanium alloy ultrasonic introduction device. The results showed that ultrasonic vibration treatment can improve the solidification structure of aluminum alloy and it can also degas, reduce microscopic loosening, refine grains, and improve the effect of improve micro-segregation. Wang Jianwu et al. [10] applied an ultrasonic wave with a power of 179 W, a frequency of 19 kHz, and a sound wave intensity of 153.33 W/cm^2^ to a 7050 aluminum alloy melt, with a treatment time of about 3 min. The experimental results showed that the microstructure of an ingot that has not been treated with ultrasonic is coarse dendrites with precipitated phase particles. After applying ultrasonic waves, both grains and the second phase particles of the ingot are refined, and the average grain size is reduced from 80 µm to 40 µm. Wang G et al. [11] studied the effect of the temperature range of melt when applying ultrasonic during solidification of Al-2Cu alloy on grain structure and cooling behavior. The studies show that when introducing ultrasound into the melt, grain refinement requires an appropriate amount of liquid metal overheating or sufficient preheating of the sonotrode.

Haghayeghi R et al. [12] introduced ultrasonic vibration to a melt before AA7075 high pressure die casting and found that, due to cavitation, the porosity decreased by 5%, the tensile strength and yield strength increased to 590 MPa and 502 MPa respectively, and the elongation increased to 18%. They pointed out that ultrasonic casting is feasible for industrial-scale applications. 

There are many studies on the ultrasonic casting of aluminum alloy, but most of them are limited to laboratory research, and the ultrasonic conduction tools used are straight-rod sonotrodes. When applying ultrasonic waves, the heat radiated by the melt will seriously affect the stability of the ultrasonic vibration system. Moreover, the titanium alloy sonotrode is prone to erosion during the casting process [13], and it is impossible to stably conduct ultrasonic waves into the molten metal for a long time. Therefore, ultrasonic-assisted casting technology has not been applied in the industry so far. In order to solve this problem, an L-shaped ceramic ultrasonic introduction device was used to introduce ultrasonic waves into the melt in the 2219 aluminum alloy casting process. The L-shaped ceramic ultrasonic introduction device can avoid the direct heat radiation of the high-temperature melt to the transducer. The ceramic sonotrode can also overcome the problem of cavitation erosion caused by the high-temperature aluminum melt and improve the stability of the ultrasonic vibration system. Through experiments without ultrasonic casting, straight-rod ultrasonic-assisted casting, and L-shaped ultrasonic-assisted casting, the effects of cavitation melt processing by an L-shaped sonotrode made of high-temperature-resistant ceramic on solidifying 15t AA2219 aluminum alloy ingots were discussed.

## 2. Materials and Methods

### 2.1. Casting

Without ultrasonic casting, the 2219 aluminum alloy was proportioned according to GB/T3190-2008 standard. 15t commercial purity aluminum was put into a 20t-capacity melting furnace, heated and melted, which was then fully stirred by the electromagnetic stirrer at the bottom of the furnace. The following step was slag removal, after which metal additives like Cu and Mn were added into the aluminum melt. The refined product was then taken to degas and remove slag. After another slagging, the direct reading spectrometer was used to analyze the components, the results of which are shown in Table 1. After all components reached the standard, Al–Ti–B refiner was added. Then the melt was sent to the hot top crystallizer along a channel for casting. The casting parameters are listed in Table 2. An ingot (Named NO-UTS ingot) with a size of Φ650 mm × 6300 mm was produced and moved to the heat treatment furnace for homogenization heat treatment at the temperature of 540 °C for 60 h.

**Figure 1:**
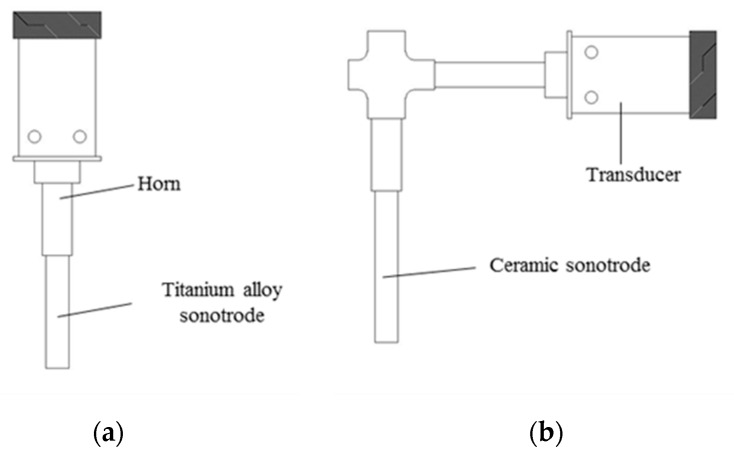
Structure of ultrasonic-introducing sonotrodes: (**a**) straight-rod sonotrode made of titanium alloy; (**b**) L-shaped sonotrode made of ceramic.

**Figure 2:**
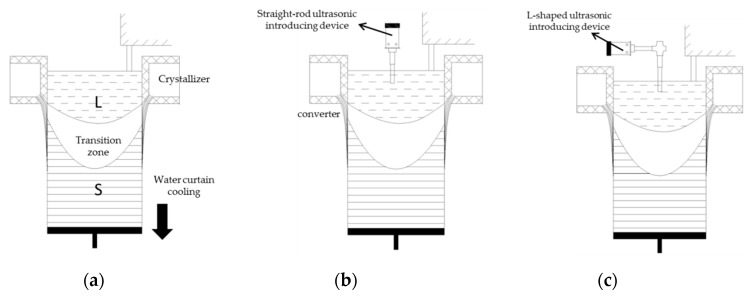
Different casting processes: (**a**) semi-continuous casting without ultrasonic treatment; (**b**) semi-continuous casting with the straight ultrasonic-introducing rod; (**c**) semi-continuous casting with L-shaped ultrasonic-introducing rod.

With ultrasonic casting, under the condition that other technological conditions are as constant as possible after casting becomes stable, the ultrasonic wave was directly introduced from the hot top. Then the straight-rod titanium alloy sonotrode and L-shaped ceramic sonotrode made by our group were used to introduce an ultrasonic wave (the structural sketches of the two ultrasonic rods are shown in Figure 1). Two different sonotrodes with a size of Φ50 mm × 185mm were inserted into the liquid surface below about 50 mm. The piezoceramic transducer was used to convert high frequency current from the generator to mechanical oscillations of the same frequency. The ultrasonic power was 800 W, the ultrasonic frequency was 23 ± 0.2 KHz, and the amplitude was 15 μm. In the final stage of casting, when the melt level dropped, the ultrasonic vibration stopped and the ultrasonic vibration system was removed. Two Φ650 mm × 6300 mm ingots (I-UTS ingot and L-UTS, separately) were cast separately (the casting device is shown in Figure 2), and the cast large ingots were transferred into the heat-treatment furnace and homogenized through the same heat-treatment process.

### 2.2. Sampling

**Figure 3:**
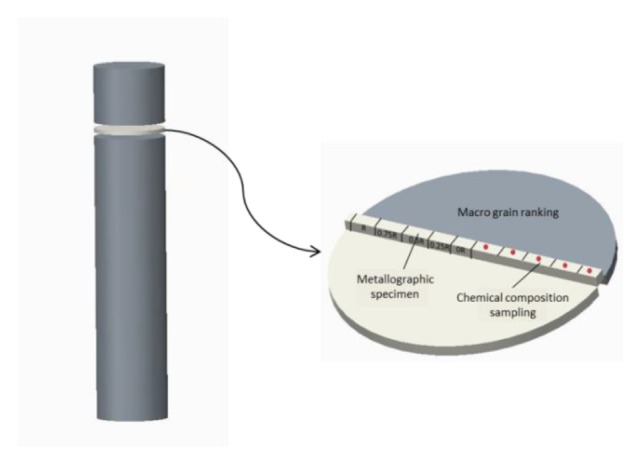
Schematic diagram.

After the 10 mm thick oxide inclusion layer was removed from the surface of three round ingots, the samples with a specification of Φ630 mm × 25 mm were cut at 500 mm from the top of the ingot. The sample was cut along the radial diameter, one part for macrostructure analysis, and the other part for microstructure analysis and chemical composition analysis. For the latter, we needed to take smaller samples at positions of 0R, 1/4R, 1/2R, 3/4R, R. The sampling locations are shown in Figure 3.

### 2.3. Characterization 

Firstly, the erosion morphology of the sonotrode of the ultrasonic-introducing device was observed after ultrasonic treatment. Then, according to the GB/T 3246.2-2000 standard, the sample taken from the ingot was polished with sandpaper, washed with alcohol, and dried. Then, it received macro corrosion and macro grain ranking. The corrodent was prepared with 10 mL of HF with 42% concentration, 5 mL of HCl with 36% concentration, 5 mL of HNO_3_ with 65% concentration, and 380 mL of water. According to the GB/T 3246.2-2000 standard, the metallographic specimen was ground and polished on an MP-2B grinding and polishing machine (Weiyi Test Equipment Manufacturing Corporation, Laizhou, China), washed with clear water, and etched with the above corrodent for 60 s. Then the metallurgical structure of the ingot was observed through an OLYCLA-DSX500(OLYMPUS Corporation, Tokyo, Japan) metallurgical microscope and the morphology, size, and distribution of its precipitation phase were observed through a Phenom automatic table scanning electron microscope (Phenom-world BV, Eindhoven, Holland). In addition, the amount of alloying element was analyzed by EDS (energy dispersion spectrometer). An Inductively Coupled Plasma Optical Emission Spectrometer (ICP-OES, Shimadzu Corporation, Tokyo, Japan) was also used to test the chemical components of different positions.

## 3. Results and Discussions

### 3.1. Anti-Corrosion Property of L-Shaped Sonotrode

**Figure 4:**
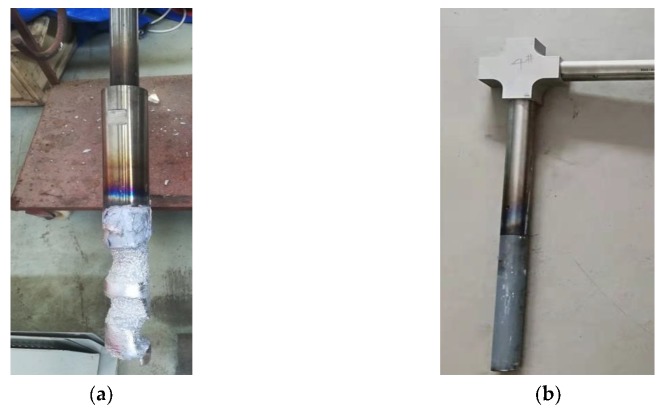
Morphology of sonotrodes: (**a**) straight-rod titanium alloy sonotrode; (**b**) L-shaped ceramic sonotrode.

The morphology of the straight-rod and L-shape sonotrode after 24 h of experiment is shown in Figure 4. It can be seen that the straight-rod titanium alloy sonotrode is seriously eroded, while the L-shaped sonotrode is basically not eroded. The titanium alloy sonotrode selected in this experiment is made of titanium alloy TC_4_, which can withstand a temperature of 1700 °C and conduct ultrasonic waves efficiently, and has the advantages of excellent corrosion resistance, low density, high specific strength, and good toughness. However, in the process of introducing ultrasound, it was slowly eroded, mainly due to the formation of alloy between the titanium alloy sonotrode and metal melt [14,15], indicating that although a titanium alloy sonotrode can withstand high temperature, it cannot withstand the melting corrosion effect of metal melt. The L-shaped ultrasonic introduction device is composed of a transducer, an L-shaped combined horn, and an upright ceramic sonotrode [16] (Figure 1b). The L-shaped combined horn is composed of a first-stage horizontal horn and a second-stage vertical horn, the first-stage horn is a straight rod that propagates longitudinal mechanical vibration, and the second-stage horn is a straight rod that propagates lateral mechanical vibrations (as shown in Figure 1b). The material used for the L-shaped combined horn is titanium alloy. The material used for the sonotrode is a special ceramic with good cold and thermal shock resistance and high strength at high temperature, and a melting point that is higher than 1900 °C. It is not oxidized below 1200 °C, the protective film formed at 1200–1600 °C can prevent further oxidation, it can resist vibration and thermal shock, and cannot form alloys with metal melts [17]. Therefore, the sonotrode will not be eroded by the melt. In addition, the L-shaped combination keeps the ultrasonic transducer away from the high-temperature metal melt during operation, effectively avoiding the direct thermal radiation of the high-temperature heat flow to the transducer, thus prolonging the life of the ultrasonic transducer while the energy of the ultrasonic is continuously inserted into the metal melt.

The performance of sonotrode plays a key role in the effect of ultrasonic treatment, and the performance of sonotrode mainly depends on two main output parameters: resonance frequency and displacement amplitude. In order to compare the performance of straight-rod sonotrode and L-shaped sonotrode, it is necessary to measure the resonant frequency and output displacement amplitude of two ultrasonic introduction systems. The output amplitude of the ultrasonic vibration system used in this experiment is relatively small, so the Laser Doppler Velocimeter (LDV) is used to measure it. As shown in Table 3, the maximum amplitude and resonance frequency at the end face of the L-shaped sonotrode are 15.21 μm and 23.08 KHz, respectively. The maximum amplitude and resonance frequency at the end face of the straight-rod sonotrode are 11.67 μm and 23.13 KHz respectively. It can be seen that the ultrasonic introduction effect of L-shaped sonotrode is better than that of straight-rod.

### 3.2. Effects on Macrostructure and Microstructure

**Figure 5:**
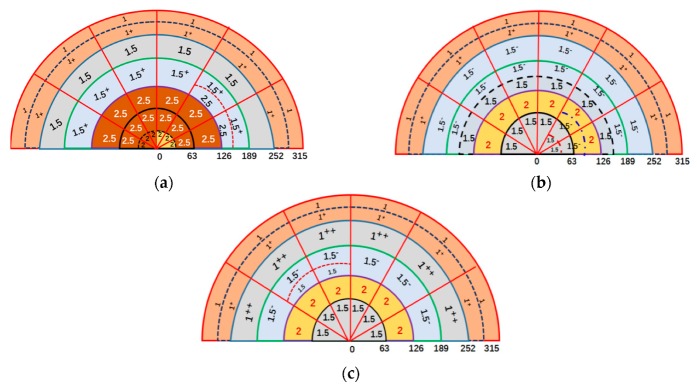
Grain grade diagram of ingots: (**a**) NO-UTS; (**b**) I-UTS; (**c**) L-UTS.

Figure 5 displays the macro grain grade diagram of the 2219 aluminum alloy ingots under three different casting processes. The macro grain size grade of the NO-UTS ingot was 1–1^+^ grade in the edge, 1.5–2.5 grade in the middle, and 2.5–2 grade in the center. The macro grain size grade of the I-UTS ingot was 1–1^+^ grade at the edge, 1.5 grade in the middle, and 1.5 grade in the center. The macro grain size grade of L-UTS ingot was 1–1^+^ grade at the edge, 1–2 grade in the middle, and 1.5 grade in the center. The macro grain size of the three ingots all shared the characterization of being smallest at the edge, largest in the middle, and smaller in the center. As for the smallest size at the edge, the reason is that when the liquid aluminum flows into the crystallizer, the temperature of the die wall is very low due to the action of cooling water. As the undercooling of the edge area increases, resulting in a large number of nucleation, the die wall can be used as the starting point of nucleation to form a dense and fine equiaxed crystal zone. At the same time as the formation of the equiaxed crystal zone, the temperature of the equiaxed crystal zone increases, and the latent crystal of the equiaxed crystal zone releases the latent heat of crystallization. The cooling rate of the remaining liquid is reduced, and the degree of undercooling is reduced, so that the grain size of the middle region is larger; the grain size in the center gets smaller than in the middle because the flow of melt brings some un-melted impurities or broken dendritic crystals to the center of the ingot and heterogeneous nucleation forms thereby, though the cooling rate in the central region is slower and the temperature difference is smaller [18,19]. The grain size grade of the ingot without ultrasonic treatment was large, and the grain size grade of each position was quite different. After ultrasonic treatment with L-shaped sonotrode, the macroscopic grain size grades in the middle and the center were significantly reduced, which can effectively refine and homogenize the grain structure, thus improving the macro-grain structure of the ingot, with a better grain refinement effect than that of the straight-rod sonotrode.

**Figure 6:**
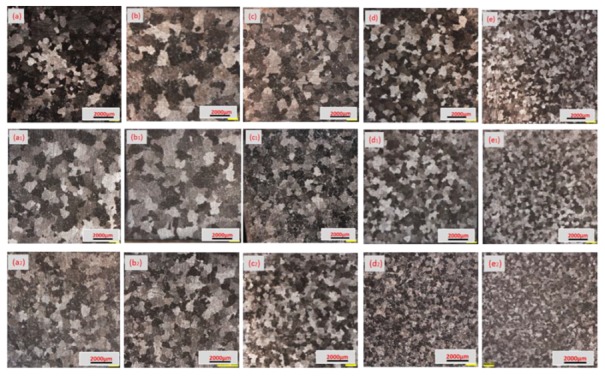
In the regions of the three ingots: (**a**–**e**) positions of 0R, 0.25R, 0.5R, 0.75R, R of the NO-UTS ingot; (**a_1_**–**e_1_**) positions of 0R, 0.25R, 0.5R, 0.75R R of I-UTS ingot; (**a_2_**–**e_2_**) positions of 0R, 0.25R, 0.5R, 0.75R, R of the L-UTS ingot.

Microstructure in the radial regions of ingots is shown in Figure 6. It can be seen from the figure that the grain size of the NO-UTS ingot is coarse and uneven, and the grain structure has been obviously refined by ultrasonic treatment. The grain size of L-UTS ingot is refined remarkably in all radial positions, and the grain size distribution is more homogenized. Compared with the I-UTS ingot, the grain structure is finer in the regions of 0.5R–0.75R. The grain size of all ingots increased first and then decreased along the radial direction, which is consistent with the macroscopic grain rating of the ingot in the above figure.

**Figure 7:**
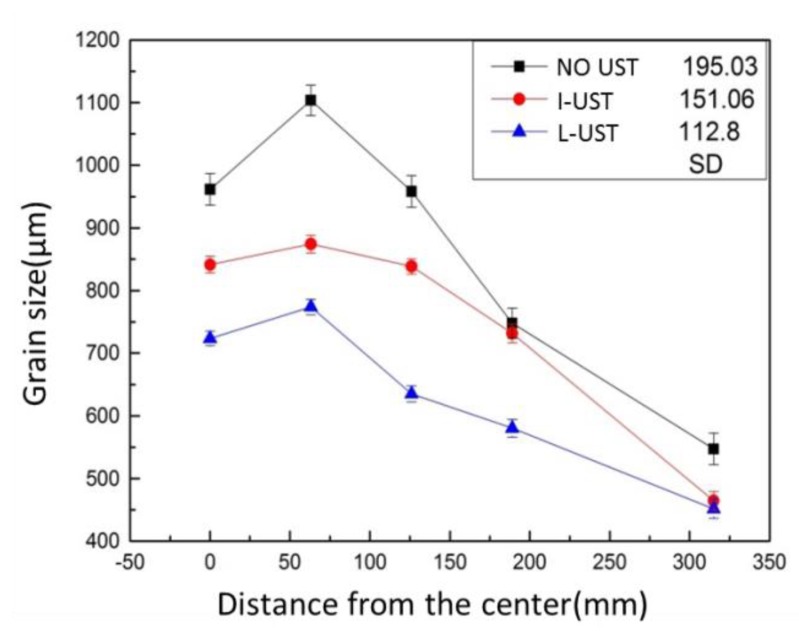
Of grains in the radial direction of the three ingots.

The average grain sizes in radial direction of the three ingots are shown in Figure 7. Compared with the NO-UTS ingot, the grain size of the L-UTS ingot reduced from 547−1100 μm to 464–874 μm, its average size decreased from 864.2 μm to 750.1 μm, and SD (standard deviation) decreased from 195.23 to 151.06. Compared with the I-UTS ingot, the L-UTS ingot had a better refining effect and produced a finer ingot with a grain size of 451–771 μm, an average grain size of 632.74 μm and SD of 112.8.

The cavitation and sound flow effects of ultrasonic during the ultrasonic casting process are the main factors affecting the solidification process of aluminum melt. The application of ultrasonic vibration in the aluminum melt can significantly increase the vibration frequency and energy of nucleus and produce a large number of cavitation bubbles in the melt. When the cavitation bubbles are resonated by ultrasound, the vibration frequency and energy of the nucleus will be significantly increased. The nucleus vibration can inhibit the growth of grains, which is conducive to the formation of equiaxed crystals and dendrites. The dendrites are also broken by vibration to form new crystal nuclei, so that the nucleation rate is improved and the crystal grains are refined. In addition, when cavitation bubbles collapse due to sound intensity, the strong shockwave will also break the coarse dendrites, and, under the stirring action of the sound flow, the broken dendrites disperse into the melt and become effective nucleation particles, so that the nucleation rate is improved and the grain size distribution is more homogenized. Finally, the strong stirring effect of ultrasound can increase the particle diffusion rate, make the solute in the melt more homogenized, and reduce the component undercooling in the front of crystallization. Small component undercooling can increase the liquidus temperature of the aluminum solution, increasing the effective undercooling and the nucleation rate, and significantly refining the grain size [20,21,22,23].

The cavitation threshold is a quantity indicating the difficulty of cavitation in the melt. The ultrasound cavitation threshold is related to many factors. In the same melt, it is closely related to the frequency, waveform and waveform parameters of ultrasound, and it increases with an increase of ultrasonic power. In the L-shaped ultrasonic introduction device, the primary and secondary titanium alloy horns are combined in an L shape, which converts the longitudinally transmitted sine wave into a laterally transmitted distortion wave, so that the mechanical vibration and sound flow effect of the ultrasonic wave in the melt are strengthened [24]. As a result, the threshold of cavitation is smaller, cavitation can occur more easily, and the grain size is smaller and more uniform than that of a straight-rod ultrasonic introduction device.

### 3.3. Effects on the Secondary Phase

**Figure 8:**
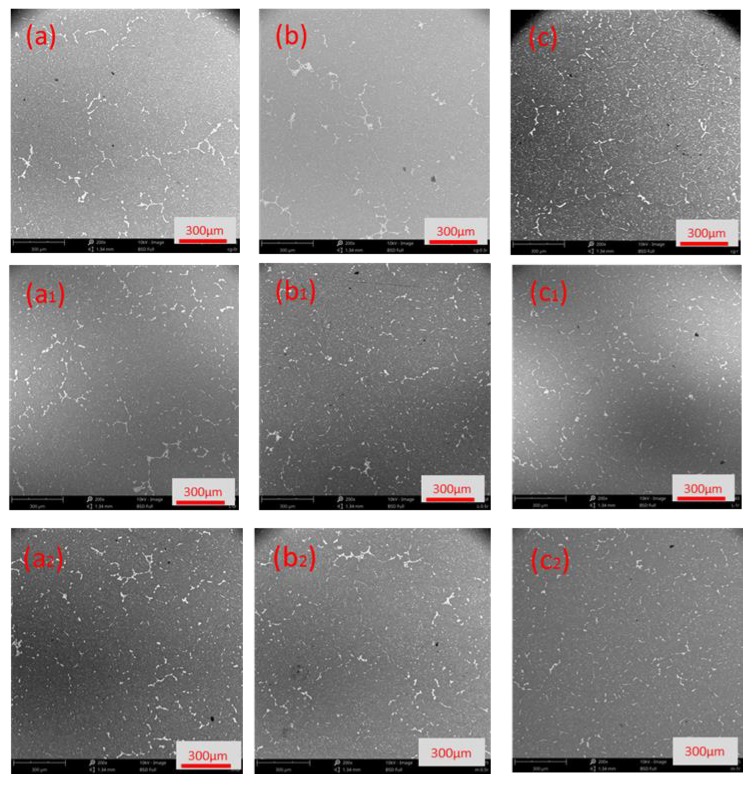
Phases of ingots under different processing parameters: (**a**–**c**) positions of 0R, 0.5R, R of NO-UTS ingot; (**a_1_**–**c_1_**) positions of 0R, 0.5R, R of I-UTS ingot; (**a_2_**–**c_2_**) positions of 0R, 0.5R, R of the L-UTS ingot.

**Figure 9:**
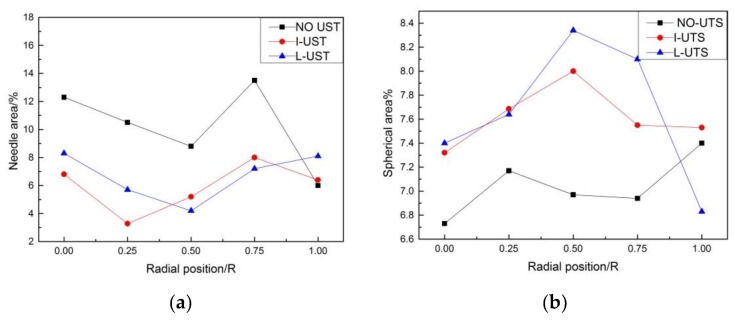
Of precipitated phase areas in different shapes: (**a**) needle area; (**b**) spherical area.

Semi-continuous casting produces large casting stress which should be eliminated through homogenization annealing to prevent cracking in subsequent mechanical processing [25]. In addition, it can promote the re-melting of the low-melting eutectic phase in the alloy to some extent, eliminate or inhibit the inhomogeneity of the microstructure and chemical composition in the grain, and increase the solid solubility of the alloying elements in the matrix so the strength of the alloy is improved [26]. The SEM images of different positions of the three ingots are shown in Figure 8. The gray area is the α-Al matrix of the ingot and the white area is the precipitation phase. The morphology of precipitated phases in ingots is needle-shaped, and granular and spherical, needle-like precipitates were most common in the NO-UTS ingot, followed by the I-UTS ingot, and least in the L-UTS ingot (as shown in Figure 9). The needle-like precipitates tend to have sharp edges and corners, which tend to cause stress concentration at the tip, which is not conducive to the plastic deformation of the matrix. The symmetrical, smooth spheroidal precipitation has a small splitting effect on the matrix, which is beneficial to the uniform plastic deformation of the matrix. Therefore, the L-UTS ingot is most conducive to the subsequent processes of the ingot. In addition, the size and distribution of the precipitated phase of the NO-UTS ingot are uneven as the precipitated phases are larger from the center to the position of 1/2R while becoming small at the edge, and an enrichment phenomenon occurs in some areas. This problem is improved partly by I-UTS casting while the precipitated phase at the position of 1/2R is still large and the enrichment phenomenon occurs. However, the precipitated phases are not only generally refined but also become more evenly distributed in the L-UTS ingot.

**Figure 10:**
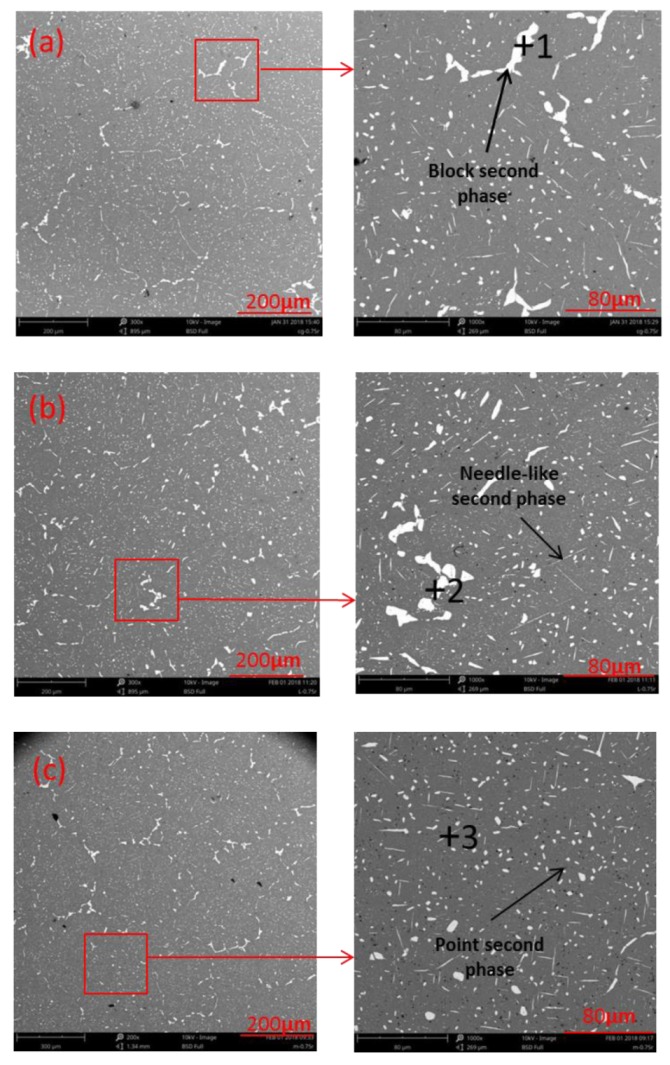
Secondary phases of ingots at the position of 0.75 R under different processing parameters: (**a**) NO-UTS; (**b**) I-UTS; (**c**) L-UTS.

The distribution of the secondary phase at the position of 0.75R in the crystal of three kinds of ingots is shown in Figure 10. For the NO-UTS ingot, the secondary phases in the crystal were mainly block-shaped and needle-shaped, with an average size of 4.1 µm. For the I-UTS ingot, the secondary phases were mainly block-shaped and dot-shaped, with an average size of 3.15 µm. For the L-UTS ingot, the secondary phases were mainly dot-shaped, with an average size of 2.38 µm. It can be seen that the secondary phase of the whole section was obviously refined by applying ultrasonic, and the agglomeration and growth of the secondary phase were effectively controlled. The energy spectrum analysis of the secondary phase in the crystal shows that the atomic number ratio of Al to Cu is 2:1 (as shown in Table 4), which indicates the secondary phase was Al_2_Cu. The effect of high-speed acoustic streaming generated by ultrasonic could stir the metal melt to some extent, thus promoting the diffusion and solid solution of alloy elements, reducing the segregation of alloy elements, and strengthening the matrix [27].

### 3.4. Effects on Macro-Segregation

**Figure 11:**
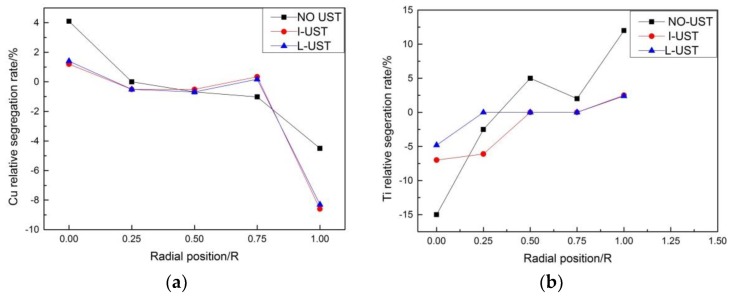
Segregation rate: (**a**) Cu; (**b**) Ti.

The degree of macro-segregation of the solute can be characterized by segregation rate S, which is obtained by the difference between ΔC_max_ (the maximum relative segregation rate) and ΔC_min_ (the minimum relative segregation rate), wherein the relative segregation rate is ΔC = (C_i_ − C_0_)/C_0_, C_i_ the concentration percentage of the element measured at each position, and C_0_ is the initial concentration percentage of the element. ΔC > 0 represents positive segregation while ΔC < 0 represents negative segregation [28]. We sampled by drilling five holes in a radial direction (Figure 3) and carried out a chemical composition test so as to know the solute element distribution along the radial direction on the cross-section of the round ingot. The distribution diagram of the relative segregation rate of Cu and Ti in 2219 aluminum alloy is shown in Figure 11, taking the radial direction as the x-axis and the relative segregation rate ΔC as the y-axis.

There was an obvious negative segregation at the edge and positive segregation at the center in all aluminum ingots in the three trials. This phenomenon is caused by the solidification rule of 2219 aluminum alloy. The relative segregation rate of Cu of the NO-UTS ingot fluctuates greatly, that is, the concentration of Cu fluctuates greatly along the radial direction and a great difference of concentration between the center and the edge could be seen. Although the uneven distribution of Cu along the radial direction was improved after ultrasonic is applied, the negative segregation of Cu reduced due to a higher concentration at the edge while the positive segregation of Cu decreased due to lower concentration at the center. The segregation rates of Cu in the I-UTS ingot and L-UTS ingot were quite the same. It is calculated that S = 0.127 in NO-UTS, S = 0.057 in the I-UTS ingot, and S = 0.058 in the L-UTS ingot, and the segregation rate of Cu was decreased by 54.3% with L-shaped ultrasonic casting. These results illustrate that ultrasonic-assisted casting can significantly reduce the macro-segregation of Cu in 2219 large aluminum alloy ingot.

AlTiB wire alloy is a common grain refiner for aluminum alloy casting, and its refinement mechanism is explained by the double nucleation theory, which asserts that TiB_2_ particles with thin TiAl_3_ layers act as nuclei in crystallization. Thus, the grain refinement properties of solid aluminum alloys depend largely on the size and shape of TiB_2_ particles and TiAl_3_ phases in their microstructures [29,30]. From the relative segregation rate of Ti element at different positions along the radial direction (Figure 11), it can be seen that the Ti element in the NO-UTS ingot had a larger positive segregation at the edge and larger negative segregation at the center. This is because Ti element will undergo a peritectic reaction with aluminum, and the segregation rate shows a tendency opposite to that of Cu element. The segregation rates and fluctuation significantly reduced after the melting of casting aluminum were treated by ultrasound. This is because the local high temperature and high pressure generated by the ultrasonic accelerates the dissolution of the coarse TiAl_3_ particles, and the ultrasonic flow agitation increases the diffusion distance of the solute Ti and uniformly distributes it in the aluminum solution. The negative pressure generated by the cavitation effect can increase the surface energy of TiB_2_ particles by removing gases, impurities, and oxides on the particle surface, thus improving the wettability between TiB_2_ particles and aluminum melt [31]. The local high temperature and high pressure produced by cavitation effects also reduce the surface tension of aluminum melt, which can further improve the wettability between particles and melt. Meanwhile, the stirring by acoustic streaming can also boost a more homogenized distribution of TiB_2_ particles in the melt [32,33]. Thus, the thin TiAl_3_ layer on the surface of TiB_2_ particles is formed and a more homogenized and finer grain structure is obtained [34,35].

## 4. Conclusions

1. The L-shaped ceramic ultrasonic introduction device can effectively avoid the erosion of high-temperature melt on sonotrode and the heat radiation of the high-temperature heat flow to the transducer.
2. The ultrasound introduced by the L-shaped ultrasonic introduction device made of ceramic can refine grains better and make their size and macro distribution more homogenized than the straight-rod ultrasonic introduction device due to the stronger mechanical vibration and sound flow effect in the melt.
3. L-shaped ultrasonic-assisted casting can produce more dot-shaped and globular precipitation phases conducive to plastic deformation of the matrix and make the distribution of precipitated phases more homogenized with less concentration than straight-rod ultrasonic-assisted casting.
4. Both ultrasonic-assisted castings can decrease macro-segregation of the chemical composition in the AA2219 aluminum alloy ingot, causing the concentration of Cu and Ti to distribute more evenly in the whole section.

## Figures and Tables

**Table 1:** Chemical composition of 2219 aluminum alloy (wt. %).

Chemical Composition	Cu	Mn	Fe	Ti	V	Zr	Si	Al
NO-UTS	5.9	0.36	0.018	0.04	0.07	0.11	0.024	Bal
I-UTS	5.91	0.35	0.02	0.04	0.07	0.11	0.025	Bal
L-UTS	5.9	0.34	0.019	0.04	0.06	0.11	0.023	Bal

**Table 2:** Casting parameters of aluminum alloy ingot.

Specification/(mm × mm)	Pouring Temperature/°C	Cooling Temperature/°C	Speed of Water Flow/(L/min)	Speed of Introducing Ingot/(mm/min)
Φ650 × 6200	696	29.1	321	24

**Table 3:** Actual output amplitude and resonance frequency of different ultrasonic vibration systems.

Type	Amplitude/µm	Frequency/KHz
**I-shaped**	15.21	23.08
**L-shaped**	11.67	23.13

**Table 4:** The mole fraction of the second phase in different ingot crystals (%).

Testing Point	Al	Cu
**1**	64.33	35.67
**2**	63.17	36.83
**3**	64.02	35.98
